# Recollections, reflections, and revelations: ethnobiologists and their “First Time” in the field

**DOI:** 10.1186/1746-4269-9-12

**Published:** 2013-02-20

**Authors:** Justin Nolan, Andrea Pieroni

**Affiliations:** 1 Department of Anthropology, University of Arkansas, Fayetteville, AR 72701, USA; 2 University of Gastronomic Sciences, Piazza Vittorio Emanuele 9, 12060, Bra/Pollenzo, Cuneo, Italy

## 

For nearly a century, ethnobiologists have collaborated with local community members in their efforts to document and safeguard our planet’s rich and varied biocultural heritage. Work in ethnobiology and ethnomedicine, including ethnobotany, ethnozoology, and ethnoecology, necessarily entails meticulous and rigorous systematic observation of the myriad ways indigenous and local communities cognize, utilize, and classify the floral and faunal resources on which they depend for survival. By its very nature, ethnobiology synthesizes humanism, ethnoscience, and empirical data collection and hypothesis-testing. Clearly, ethnographic and methodological flexibility are critical historically to the present and future successes of the programs we create and carry forth into the world.

The practice of ethnobiology is itself inevitably capable of advancing pedagogies with real and lasting applications for students with only cursory knowledge of first-hand field experiences. Medical ethnobiology and ethnobotany, including ethnopharmacology, ethnoecology, conservation biology, and their related fields and approaches, are poised to evaluate carefully the richly and distinctly expressive component of our projects, wherever they are located geographically and situated culturally. Personal experiences, narratives, and reflections are occasionally minimized to varying degrees in the now-expansive body of ethnobiological and ethnomedical literature. Although notable exceptions certainly exist, our goal in the next months is to expand the scope of dialogue by turning attention to the most engaging forms of ethnography within ethnobiological accounts--that is, we seek to capture the stories, chronicles, imaginations, and memoirs of ethnnobiologists.

Every few months the *Journal of Ethnobiology and Ethnomedicine* will begin to offer editorials drafted by several international ethnobiologists, who report their first-hand recollections of their earliest field experiences. These, we believe, are uniquely capable of rendering interpersonal interactions comprehensible and meaningful in vastly innovative, sometimes subtle, and often unexpected ways for students who are new to the field especially, and for intermediate and seasoned scholars with vested interests in the well-being of the communities they study.

In introducing this novelty within the *Journal of Ethnobiology and Ethnomedicine*, our goal is to ready readers for the array of voices, narratives, admissions, tribulations, engagements, and experiences of scholars whose works reveal how ethnobiological research is personalized and internalized, how friendships are forged, how emotions, responses, and senses are heightened in the process, and how casual and intimate field experiences coalesce and emerge through the lives and minds of scholars working early in their careers.

Readers with established research programs in ethnomedicine and ethnobiology will find the essays provocative and revealing. Undergraduate and graduate students from various programs worldwide may find these recollections compelling, inspiring, illuminating, and encouraging. We also hope they speak to the curiosities and mysteries associated inextricably with “first time encounters” in field settings. In publishing this series of essays in the *Journal of Ethnobiology and Ethnomedicine* we intend to provide readers a vibrant portrait of productive and rewarding learning journeys in the growing disciplines of ethnobiology and ethnomedicine.

